# Impact of Lipid Source on Protein Digestion and Absorption in Skimmed Goat Milk and Associated Intestinal Oxidative Stress Responses in a Caco-2 Cell Model

**DOI:** 10.3390/foods15071200

**Published:** 2026-04-02

**Authors:** Haiyan Xue, Bowei Ding, Baoyuan He, Jun Ma, Yanhui Lian, Wenmin Dong

**Affiliations:** 1 College of Food Science and Engineering, Shaanxi University of Science and Technology, Xi’an 710021, China; xuehaiyan@sust.edu.cn (H.X.); dingbowei_2027@163.com (B.D.);; 2 College of Bioresources Chemical & Materials Engineering, Shaanxi University of Science and Technology, Xi’an 710021, China

**Keywords:** goat-milk-based infant formula, goat milk protein, lipid, in vitro simulated digestion, bioactive peptides, intestinal protection

## Abstract

Liquid infant formula has garnered increasing attention due to its mild thermal processing and superior retention of bioactive nutrients. Within such matrices, the lipid source is a critical determinant of protein digestion behavior, yet its influence on peptide bioavailability and intestinal homeostasis remains undefined. Given that efficient peptide absorption is vital for the systemic delivery of bioactivity in infants, understanding the lipid–protein synergy is essential for formula optimization. Moreover, excessive oxidative stress is closely associated with impaired intestinal health and developmental disorders in infants, making the regulation of oxidative stress crucial for maintaining intestinal function. The present study evaluated the effects of three distinct lipid sources—soybean oil (SM), bovine milk fat (BM), and goat milk fat (GM)—on the physicochemical stability, proteolytic digestion, peptide release, intestinal absorption, and oxidative stress modulation of goat-milk-based infant formula. An integrated approach combining physicochemical characterization, in vitro simulated infant digestion, and a Caco-2 intestinal epithelial cell model was employed. we demonstrate that all three lipids (3% *w*/*w*) formed stable emulsions with uniform spherical structures and mean particle diameters of 117–300 nm, as visualized by laser confocal microscopy. Following in vitro simulation of infant gastrointestinal digestion, the SM group exhibited the most extensive protein hydrolysis, yielding the highest total peptide content (4.28 ± 0.10 mg/mL) and generated the highest number of peptides identified by LC-MS/MS (474 types). Bioinformatic analysis predicted that peptides from all groups possess potential antihypertensive, hypoglycemic, and immunomodulatory activities. The Caco-2 monolayer cell model demonstrated that although the GM group produced fewer identified peptide species than the SM group (365 types), it achieved significantly higher intestinal peptide absorption rate (55.34 ± 1.05%). Furthermore, the GM digests provided superior protection against H_2_O_2_-induced oxidative stress in Caco-2 cells, markedly reducing reactive oxygen species levels and suppressing the expression of pro-inflammatory cytokines TNF-α and IL-6. Collectively, these findings reveal that while soybean oil promotes more extensive proteolysis, the use of homologous goat milk lipid enhances peptide bioaccessibility and confers potential cytoprotective effects on intestinal epithelial cells, underscoring its potential as a preferred lipid source in infant formula formulations.

## 1. Introduction

The gastrointestinal tract and immune system of infants and young children are immature, posing significant challenges in meeting their nutritional needs. Compared to cow’s milk, goat milk is physiologically better suited for infants because it features a lower ratio of casein to whey protein and a higher proportion of β-casein relative to α_s1_-casein. This unique protein profile closely resembles human breast milk, which significantly enhances protein digestibility and reduces allergenic potential [1]. In 2018, global goat milk production exceeded 18 million metric tons, and its output is projected to increase by approximately 53% over the next decade, indicating substantial market potential [2]. Against this background, elucidating the molecular interactions within this matrix is critical for optimizing nutritional delivery.

Lipids, the key nutrients in infant formula, contribute to ≥50% of the total energy intake of infants and young children and participate in critical physiological processes, including nervous system development, immune regulation, and metabolic homeostasis [3]. They are not merely energy sources but also structural templates that govern the digestive fate of co-ingested proteins. Existing studies have demonstrated that the interaction between lipids and proteins during the processing of dairy products can induce structural modifications in proteins [4,5], thereby influencing their digestive behavior and physiological activity.

With advances in nutritional science, it is now recognized that different nutrients in food do not function as simple additive components. Instead, their microstructure and interactions among nutrients are critical factors in regulating digestive behavior, nutrient bioaccessibility, and physiological responses [6]. In commercial goat-milk-based formulations, these lipid fractions typically comprise a strategic blend of plant-based oils (e.g., soybean oil, sunflower, or palm oil) and animal-based fats (e.g., bovine or goat milk lipids). These sources differ fundamentally in their fatty acid profiles and the stereospecific distribution of fatty acids within the triacylglycerol backbone—specifically the enrichment of palmitic acid at the sn-2 position —which critically dictate their interfacial behavior and the release of free fatty acids during digestion [7,8]. Recent evidence suggests that such structural variations also modulate the adsorption of proteins onto lipid droplet surfaces and their subsequent susceptibility to proteases [4,5], significantly influencing protein hydrolysis. For instance, the digestion rate of β-lactoglobulin (β-Lg) in whole milk is slower than that in skim milk, possibly because of the stabilizing effect of free fatty acids on β-Lg during digestion [9]. Therefore, we hypothesized that the lipid source in goat-milk-based formula differentially modulates the proteolytic patterns of proteins, thereby yielding distinct peptide profiles that exert divergent effects on intestinal absorption efficiency and subsequent cellular responses.

Peptides, as essential products of protein digestion, serve as a crucial source of amino acids for both infants and adults. With advances in nutritional science, it has been recognized that protein digestion releases specific bioactive peptides with antimicrobial, antihypertensive, hypoglycemic, immunomodulatory, and anti-inflammatory activities [10]. These bioactive peptides support the development of the intestinal barrier, enhance immune function, and play a vital role in the healthy growth, neurodevelopment, and long-term well-being of infants [11]. Current research, however, has predominantly focused on macroscopic protein digestion rates. A significant research gap remains regarding how different lipid origins (plant vs. animal) qualitatively influence the specific types of bioactive peptides released and their subsequent systemic bioaccessibility. Specifically, while some studies suggest that plant-based oils may accelerate protein hydrolysis [12], it remains unclear whether a higher degree of proteolysis necessarily correlates with superior peptide bioaccessibility or the maintenance of intestinal epithelial homeostasis. Furthermore, the potential of homologous goat milk lipids to provide superior bioactivity compared to heterologous bovine or plant-based sources remains under-explored.

Human colon adenocarcinoma-derived Caco-2 cells (Caco-2 cell line) differentiate into polarized monolayers to express the key brush border enzymes and intact junction proteins and therefore serve as a widely accepted in vitro model for evaluating intestinal absorption and epithelial barrier integrity in humans [13].

To validate this hypothesis, soybean oil, bovine milk lipid, and goat milk lipid were selected as lipid sources and homogenized with skimmed goat milk to prepare 3 stable emulsion systems. By integrating in vitro simulated infant digestion with LC-MS/MS peptide mapping and a Caco-2 cell monolayer model, we systematically evaluated the influence of lipid sources on peptide release, transepithelial transport, and cellular responses to oxidative challenges. These findings provide a molecular-level framework for the strategic selection of lipid sources to enhance the functional efficacy of goat-milk-based formulations.

## 2. Materials and Methods

### 2.1. Materials and Reagents

Fresh goat milk and bovine milk were provided by Shaanxi JinNiu Dairy Co, Ltd. (Xi’an, China). Soybean oil used in our experiment was acquired from Yihai Kerry Food Marketing Co, Ltd. (Xi’an, China). Nile Red and fluorescein isothiocyanate (FITC) were purchased from Shanghai Yuanye Bio-Technology Co., Ltd. (Shanghai, China). Pepsin, trypsin, DPPH, pancreatic lipase, and hydrogen peroxide (H_2_O_2_) were obtained from Sigma-Aldrich Co. (St. Louis, MO, USA). Caco-2 cells were supplied by Procell Life Science & Technology Co, Ltd. (Wuhan, China). Trichloroacetic acid (TCA) was purchased from Tianjin Kemiao Chemical Reagent Co., Ltd. (Tianjin, China). Dulbecco’s Modified Eagle Medium (DMEM), trypsin-EDTA solution, fetal bovine serum (FBS), 2′,7′-dichlorodihydrofluorescein diacetate (DCFH-DA) assay kit, and CCK-8 assay kit were purchased from Aorijia Precision Biotechnology Co, Ltd. (Xi’an, China). Alkaline phosphatase (ALP) assay kit was purchased from Addison Biotechnology Co, Ltd. (Zhenjiang, China). We bought interleukin-6 (IL-6) assay kit and human tumor necrosis factor-α (TNF-α) ELISA kit from Jiangsu Meimian Industrial Co, Ltd. (Yancheng, China). Bovine bile salt, formic acid and acetonitrile was purchased from Macklin Biochemical Co., Ltd. (Shanghai, China). Inorganic reagents, such as sodium chloride (NaCl) and potassium chloride (KCl), were provided by Sinopharm Chemical Reagent Co., Ltd. (Shanghai, China). All other chemical reagents used in this study were of analytical grade.

### 2.2. Preparation and Stability of Goat Milk Samples with Different Lipid Sources

Fresh goat milk was centrifuged at 5000 rpm and 4 °C for 20 min. The lower layer was collected as skimmed goat milk, whereas the upper layer was collected as milk lipid. After heating in a 65 °C water bath and subsequent filtration, the goat milk lipid was purified. Bovine milk lipid was obtained using a similar method. Soybean oil, bovine milk lipid, and goat milk lipid were dispersed in skimmed goat milk at mass fractions of 0%, 1.0%, 3.0%, and 5.0%, and were designated as SM, BM, and GM groups, respectively, which was then homogenized for 5 min at 13,000 rpm using a high-speed homogenizer(Shanghai Fluko Technology Development Co., Ltd., Shanghai, China) to prepare emulsion samples for further analysis.

#### 2.2.1. Stability of Emulsion Samples

The effects of different concentrations on emulsion stability were evaluated from four perspectives: particle size, zeta potential, microstructure, and long-term reconstitution stability, to determine the optimal fat concentration. Prior to particle size and zeta potential measurements, all samples were diluted 100-fold with PBS to reduce multiple scattering effects. The average particle size and particle size distribution were determined using a Brookhaven Omni nanoparticle analyzer (Brookhaven Instruments, Holtsville, NY, USA), with refractive indices set to 1.460 for milk emulsions and 1.333 for water. The measurements were performed at 25 °C and the equilibration time was set to 120 s. The average particle size was expressed as the intensity-weighted mean diameter. Particle size distribution was calculated by the instrument’s software. The zeta potential of the emulsions was measured using a zeta potential analyzer (Brookhaven Instruments, Holtsville, NY, USA) at 25 °C. All measurements were conducted in five replicates, and data were expressed as mean ± standard deviation.

#### 2.2.2. Laser Confocal Microscopy Observation

The microstructure of the emulsions was examined using a confocal laser scanning microscope (Nikon A1RHD25, Tokyo, Japan). The samples were stained using a combination of FITC solution (0.1%, prepared with water) and Nile Red solution (0.1%, prepared with ethanol) at a ratio of 1:3. Then, 250 µL of sample was mixed with 25 µL of the combined dye solution, and the images were captured under a 40× objective lens after excitation with a 633 nm He–Ne laser and 488 nm argon ion laser, respectively. In addition, the physical state of the solution was observed after re-dissolution and at 3 months after re-dissolution to further evaluate the emulsion stability under real-world conditions.

### 2.3. Preparation of In Vitro Simulated Infant Gastrointestinal Digestion Samples

Simulated infant gastrointestinal digestion was performed according to the in vitro digestion protocol described by Xue et al. [14]. Simulated gastric fluid (SGF: 94 mM NaCl, 13 mM KCl) and samples were mixed at a ratio of 1:1 and pre-warmed at 37 °C for 20 min. The pH was adjusted to 5.0 with dilute hydrochloric acid, followed by the addition of pepsin (400 U/mL). The mixture was incubated at 37 °C with shaking at 100 rpm for 2 h. Samples were collected at 0, 30, 60, 90, and 120 min for peptide content analysis. After digestion, the reaction was terminated by adjusting the pH to 8.0 with NaOH. Subsequently, simulated intestinal digestion was conducted by adding simulated intestinal fluid (SIF: 164 mM NaCl, 10 mM KCl, 85 mM NaHCO_3_, 3 mM CaCl_2_, 6.1 mM bovine bile salt) at a ratio of 1:1. The procedure was similar to that of simulated gastric digestion, except that the pH was adjusted to 7.0, and trypsin (64 U/mL) and pancreatic lipase (16 U/mL) were added. After digestion, enzymes were inactivated by heating in a water bath at 100 °C for 5 min. The o-phthaldialdehyde (OPA) method was used to measure the degree of protein hydrolysis after digestion, as proposed by Nielsen et al. [15], to evaluate the effects of different lipid sources on protein hydrolysis. For the peptide content at different digestion times, the hydrolysate was mixed with an equal volume of 10% trichloroacetic acid (TCA) and allowed to stand for 10 min. Subsequently, the resulting solution was centrifuged at 1800× *g* for 10 min to remove unhydrolyzed protein. The supernatant was collected, and the peptide content was determined by the biuret method [16]. The DPPH radical scavenging activities of hydrolysates at different gastrointestinal digestion times were determined with slight modification according to a previously reported method [12]. Briefly, 1 mL of methanol was added to 1 mL of hydrolysates, followed by 2 mL of 0.2 mmol/L DPPH solution. After mixing, the absorbance at 520 nm was recorded, and the value was calculated using the following Formula (1):

(1) DPPH free radical scavenging rate (%) = [1 − (A_i_ − A_j_)/A_0_] × 100%

where A_i_ represents the absorbance value of the sample reacted with DPPH solution; A_j_ is the absorbance value of the reaction between sample and methanol; and A_0_ indicates the absorbance of the DPPH solution reacted with deionized water.

### 2.4. Peptidomics Analysis of Digested Samples by LC-MS/MS

Liquid chromatography–tandem mass spectrometry (LC-MS/MS) was used to identify the peptides in the filtrate after the digested samples were collected using ultrafiltration tubes (5 kDa). The analytical system included a Thermoelectric Easy-nLC 1200 (Thermo Fisher Scientific, Waltham, MA, USA) system coupled to Orbitrap Exploris 480 mass spectrometers (Thermo Fisher Scientific, Waltham, MA, USA). Mobile phase A consisted of 0.1% (*v*/*v*) formic acid in water, and mobile phase B consisted of 0.1% (*v*/*v*) formic acid in acetonitrile. The flow rate was set at 300 nL/min, and the separation was performed using the following gradient: 0–5 min, 5% B; 5–45 min, 5–50% B; 45–50 min, 50–90% B; 50–55 min, 90% B; 55–65 min, 90–5% B. The parameters of the mass spectrometer were set according to the method previously described by He et al. [17]. The raw mass spectrometry data were converted to MGF format using MM File Conversion software (Msconvert version 2.0), followed by searching against the UniProt database with the MASCOT search engine (http://www.matrixscience.com/, accessed on 20 January 2025). Search parameters included trypsin as the protease, allowing up to two missed cleavages, with carbamidomethylation of cysteine as a fixed modification and oxidation of methionine as a variable modification. Peptides were filtered with a false discovery rate (FDR) < 1% to ensure reliability. For the bioactive peptides identified by LC-MS/MS, we further screened and classified their biological activities using BIOPEP-UWM database (https://biochemia.uwm.edu.pl/, accessed on 5 February 2025) and newly identified bioactive peptides from published studies as Supplementary Data.

### 2.5. Caco-2 Cell Culture

To evaluate the peptide absorption capacity, a Caco-2 cell model was established following the culture method described by Peng et al. [18]. Briefly, cells were cultured in DMEM medium supplemented with 20% fetal bovine serum, streptomycin (0.1 mg/mL), and penicillin (100 U/mL), with the medium replaced every 1–2 days. When the cells reached 80–90% confluence, those in the logarithmic growth phase were digested with EDTA-trypsin, centrifuged, resuspended in medium, and seeded into 6-well Transwell plates (0.4 μm pore size, polyester membrane, Guangzhou Biofil Company, Guangdong, China) at a density of 5 × 10^4^ cells/cm^2^. Prior to seeding, 1.5 mL and 2.6 mL of medium were added to the apical and basolateral chambers, respectively, and the plates were equilibrated in a 37 °C incubator for 2 h. After 21 days of culture, the cells fully differentiated into a monolayer structure with intestinal barrier function. Transepithelial electrical resistance (TEER) was measured using a trans-epithelial resistance meter to verify the integrity and permeability of the model. In addition, the cytotoxicity of the hydrolysates was determined using the Cell Count Kit-8 (CCK-8) according to the manufacturer’s instructions and the method described by He et al. [17].

#### 2.5.1. Absorption Analysis of Total Peptides in Hydrolysates

The absorption of total peptides in the hydrolysate was evaluated using Caco-2 cells. The monolayer cell membrane was washed 3 times with PBS. On the AP side of the Transwell plate, 0.5 mL of gastrointestinal digestion sample was added, whereas 1.5 mL of HBSS buffer solution was added to the BL side. After incubation at 37 °C for 2 h, the solutions from both sides were collected. The AP and BL side solutions were mixed with an equal volume of 10% trichloroacetic acid solution and allowed to stand for 10 min, followed by centrifugation at 1800× *g* for 10 min to remove high-molecule proteins. The total peptide content in the supernatant was determined using the biuret method [16]. The absorption rate of the peptides was calculated using the following Formula (2):

(2) Absorption (%) = CML_BL_/CML × 100%

where CML_BL_ indicates the transported peptide amount in the BL side (µg), whereas CML indicates total peptide amount in hydrolysate added to the AP side (µg).

#### 2.5.2. Determination of Intracellular ROS Scavenging Rate of Hydrolysates from Different Fat Sources

The protective effect of hydrolysates from different fat sources against hydrogen peroxide (H_2_O_2_) induced intestinal oxidative stress was evaluated by Caco-2 cell model. Caco-2 cells were seeded in 12-well plates at a density of 1 × 10^5^ cells/mL and incubated at 37 °C until the cell confluency reached 80%. Milk hydrolysate samples from different fat sources were added, followed by 12 h of incubation and subsequent induction with 200 μmol/L H_2_O_2_ for an additional 4 h. For the control group, an equal volume of complete medium was added for 12 h of incubation prior to induction with 200 μmol/L H_2_O_2_ for 4 h. For the blank group, cells were only incubated with an equal volume of complete medium for 16 h without H_2_O_2_ induction. After the respective treatments, all cells were washed twice with PBS and then incubated with 10 μmol/L DCFH-DA staining solution for 30 min at room temperature in the dark. Fluorescence intensity was determined using a microplate reader (Thermo Fisher Scientific, Waltham, MA, USA) with an excitation wavelength of 485 nm and an emission wavelength of 530 nm. The relative fluorescence intensity of each sample was calculated as the ratio of the measured fluorescence intensity to cell viability. Fluorescent images were captured and recorded using an inverted fluorescence microscope (Nikon Tis, Kyoto, Japan).

#### 2.5.3. Determination of Inflammatory Level

Logarithmic-phase Caco-2 cells were seeded in 12-well plates at a density of 1.5 × 10^5^ cells per well and incubated at 37 °C with 5% CO_2_ for 24 h. Different digestive products were then added, and the cells were cultured until the cell confluency reached 80%. For the experimental group, the supernatant was discarded and the cells were induced with 600 μmol/L H_2_O_2_ for 6 h to trigger inflammatory response. For the control group, cells were incubated with complete medium alone to 80% confluency, followed by 6 h of induction with 600 μmol/L H_2_O_2_. For the blank group, cells were only cultured with complete medium without H_2_O_2_ induction. The expression levels of IL-6 and TNF-α were determined using Enzyme-Linked Immunosorbent Assay (ELISA) kits (Shanghai Xinyue, Shanghai, China).

### 2.6. Statistical Analysis

All experiments were performed in three independent biological replicates. Data are expressed as mean ± standard deviation (SD). IBM SPSS Statistics 29.0.2 software was used for statistical analysis. One-way ANOVA was applied to compare differences among groups, followed by LSD post hoc tests. Differences were considered statistically significant at *p* < 0.05. Graphical visualization was performed using Origin 2021.

## 3. Results and Discussion

### 3.1. Effects of Different Lipid Concentrations on the Stability of Goat Milk Emulsion

**Figure 1:**
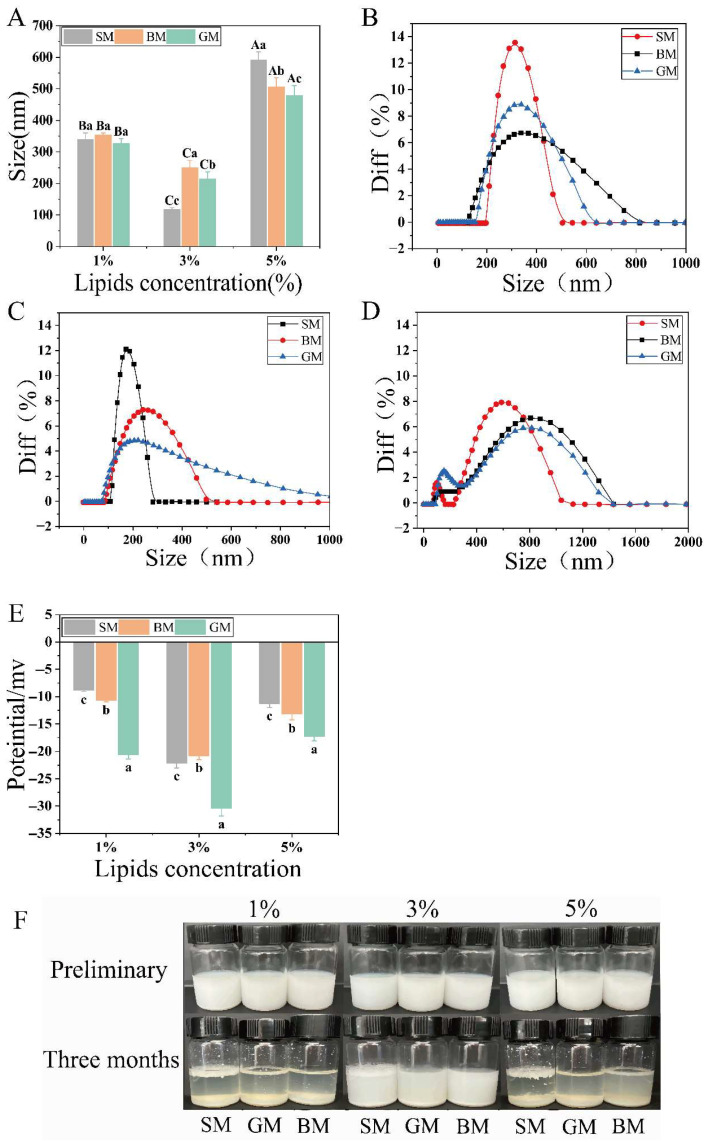
(**A**) Effects of different lipid sources on the mean particle size of milk emulsions; (**B**) Effect of 1% lipid concentration on distribution of particle sizes in milk emulsions; (**C**) Effect of 3% lipid concentration on the particle size distribution of milk emulsions; (**D**) Effect of 5% lipid concentration on the particle size distribution of milk emulsions; (**E**) Effects of different lipid sources on the zeta potential of milk emulsions; (**F**) Storage stability of different milk emulsions after 0 and 90 days of storage. (Different letters indicate *p* < 0.05).

The aggregation of different components in the emulsion leads to system instability, affecting the effective contact between enzymes and proteins [19,20]. As shown in Figure 1A, when the concentration of lipid was 1%, the average emulsion particle size ranged from 327 to 354 nm, showing no significant differences in emulsion among all groups (*p* > 0.05). When the lipid concentration was increased to 3%, the average emulsion particle size (Figure 1A) decreased to 117–300 nm, and the particle size distribution shifted leftward (Figure 1C). This reduction in particle size effectively mitigated creaming and sedimentation of large lipid droplets, thereby significantly improving the colloidal stability of goat milk. When the lipid concentration reached 5% (Figure 1D), all 3 samples exhibited broad, heterogeneous bimodal particle size distributions accompanied by a significant increase in particle size (*p* < 0.05), which may affect emulsion stability and increase the risk of phase separation or droplet coalescence [21]. Previous studies have shown that under high-fat conditions, the increased number of droplets leads to a higher collision probability. Proteins on the droplet surface aggregate via intermolecular interactions, reducing the percentage of adsorbed proteins at the oil-water interface, thereby promoting the formation of large droplets and adversely affecting emulsion stability [22,23]. More importantly, excessive droplet aggregation and coalescence may restrict the interaction between proteases and protein substrates, thereby affecting the reliability of subsequent protein digestion analysis.

The zeta potential values showed a similar concentration-dependent trend. As shown in Figure 1E, at 3.0% lipid concentration, the absolute value of the emulsion potential reached the highest, with the GM group exhibiting the highest absolute zeta potential (30.4 ± 1.4 mV; *p* < 0.05), indicating greater emulsion stability and reduced droplet aggregation, which is beneficial for subsequent in vitro digestion. To better reflect emulsion stability under real-world conditions, milk samples containing varying lipid contents were stored at 4 °C for stability monitoring. Initially, all freeze-dried milk samples demonstrated good re-solubility and no aggregation. However, after 3 months of storage, only emulsions containing 3% lipid maintained relatively stable performance, showing superior long-term stability compared with other concentrations.

**Figure 2:**
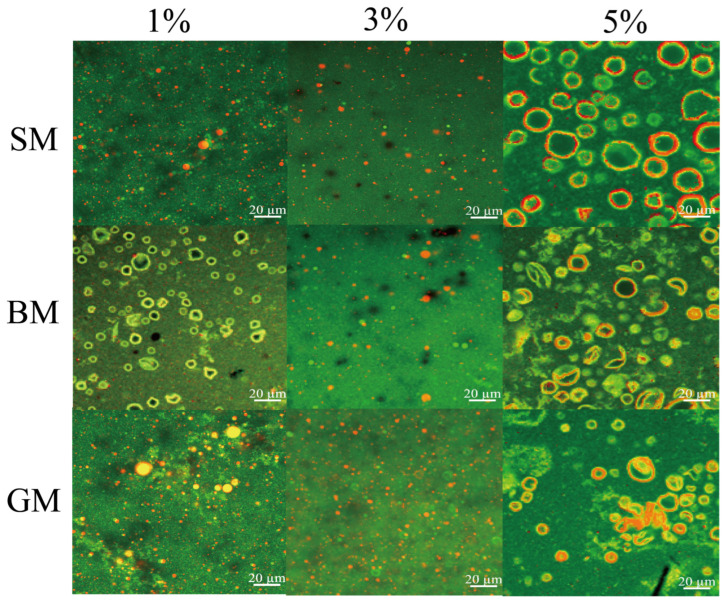
Microstructure of different milk emulsion samples. The scale bar = 20 μm.

Figure 2 illustrates the structure of goat milk lipid globules with varying lipid concentrations observed by laser confocal microscopy. At 3% lipid concentration, sufficient emulsifier molecules in the aqueous phase stabilized the droplets, and the 3 emulsion samples formed smaller, uniform spherical particles, which was consistent with particle size measurements. In contrast, when the lipid content reached 5%, severe droplet aggregation and irregular large droplets were observed, which was consistent with the structural characteristics of coconut milk fat globules at different fat concentrations reported by Chen et al. [22]. This microscopic evidence further confirmed that 3% was the optimal lipid concentration to maintain a stable emulsion system, which could exclude the interference of system instability on protein digestion and ensure the accuracy of subsequent in vitro digestion and cell experiments.

### 3.2. Effects of Different Lipids on Simulated Digestion of Emulsion in Gastrointestine

**Figure 3:**
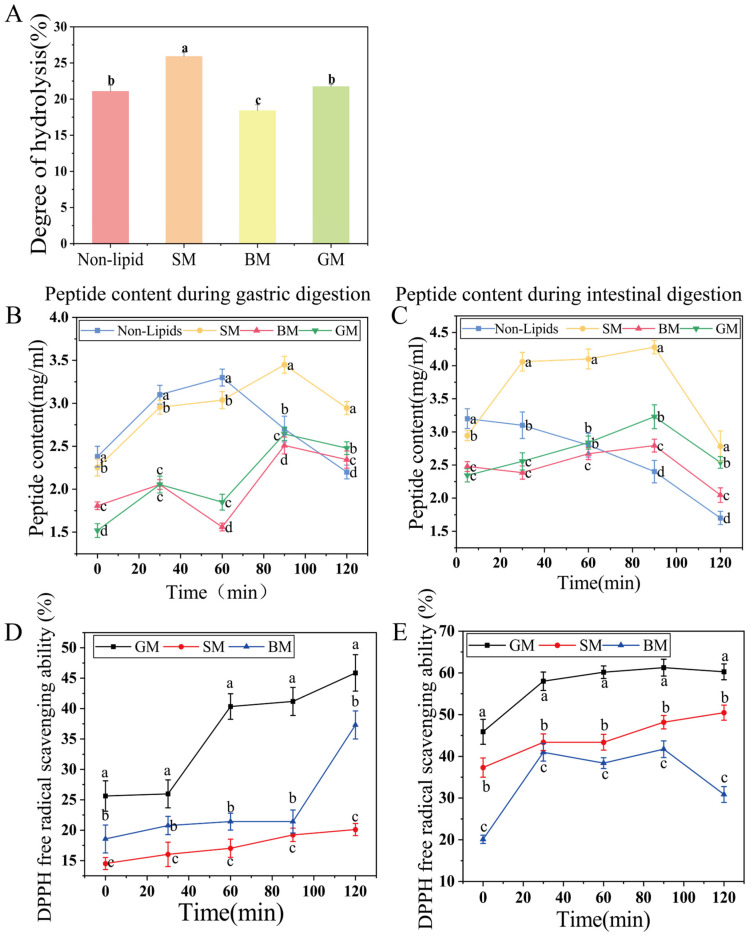
(**A**) Effects of different lipids sources on digestive hydrolysis. (**B**) Effects of different lipid sources on peptide content in samples during simulated gastric digestion. (**C**) Effects of different lipid sources on peptide content in samples during simulated intestinal digestion. (**D**) Effects of different fat sources on DPPH radical scavenging activity of samples after simulated gastric digestion. (**E**) Effects of different lipid sources on DPPH radical scavenging activity of samples after simulated intestinal digestion. (Different letters indicate *p* < 0.05).

We employed an in vitro infant digestion model to evaluate the effects of different lipid sources on the digestion of goat milk proteins. Figure 3A shows the degree of protein hydrolysis after simulated digestion in infants and young children, the overall DH is significantly lower than that observed in typical simulated adult digestion of dairy products. In simulated infant digestion, the gastric pH is 5, which is higher than the optimal pH for pepsin. Meanwhile, proteins cannot be fully denatured at this pH, thereby reducing the accessibility of protease to cleavage sites and consequently decreasing the degree of hydrolysis. The results revealed significant differences in the hydrolysis of milk samples from different lipid sources (*p* < 0.05). The SM group exhibited the highest protein hydrolysis degree (25.93 ± 0.53%; *p* < 0.05) at the end of simulated digestion, followed by the GM group (21.88% ± 0.28%). Although hydrolysis in the GM group was lower compared with the SM group (*p* < 0.05), it was still significantly higher than in the BM (*p* < 0.05), indicating that lipids with different physicochemical properties exhibit distinct regulatory effects on the hydrolysis of goat milk proteins. This finding contrasts with the report by de Figueiredo Furtado et al. [24], who observed that the proteolysis of milk proteins was not affected by lipid composition. However, their assessment of proteolysis was based solely on SDS-PAGE, whereas a key finding of our study is that lipid composition significantly influences the digestion of goat milk proteins, particularly in terms of the degree of hydrolysis.

Figure 3B,C illustrate the peptide content during simulated gastric digestion and simulated intestinal digestion in infant and young children, respectively. In skimmed milk (Non-Lipids in Figure 3B,C), complete lipid removal resulted in direct exposure of casein and whey proteins to pepsin, producing a rapid increase in peptide content within 60 min, followed by a gradual decline. After 90 min of gastric digestion, the peptide content in the SM group reached the highest level of 3.45 mg/mL, significantly higher than in the skimmed goat milk group (*p* < 0.05). This effect may be attributed to the higher contents of unsaturated fatty acids present in soybean oil, which form a more flexible structure at the water–oil interface, inducing changes in protein conformation, thereby making the hydrophobic regions and enzyme cleavage sites more accessible to proteases and enhancing enzymatic efficiency [25,26]. After entering intestinal digestion (Figure 3C), the peptide content in the skimmed goat milk group continuously decreased. The peptide content in the SM group was significantly higher than in the BM and GM groups (*p* < 0.05). After 90 min of intestinal digestion, the peptide content in the SM group reached the peak of 4.28 ± 0.10 mg/mL. The emulsion with added lipids formed chylomicron-like structures during intestinal digestion, which reduced the contact between peptides and digestive enzymes such as trypsin, thereby decreasing the degradation rate of peptides by trypsin. Compared with the BM and GM groups, the SM group exhibited smaller particle sizes, which promote the dispersion of lipids and disrupt the protein network in the clot, thus yielding a looser, more porous curd and making protein substrates more accessible to digestive enzymes, finally accelerating the hydrolysis process of proteins [27].

As shown in Figure 3D,E, all 3 emulsion groups exhibited an overall upward trend in DPPH radical scavenging activity during digestion. This finding suggests that goat milk could generate small-molecule substances with antioxidant activity after hydrolysis, such as low-molecular-weight peptides and amino acids, thereby enhancing the scavenging efficiency of digestion products towards DPPH radicals. Notably, the GM exhibited the highest antioxidant capacity throughout the digestion process (*p* < 0.05), achieving a DPPH scavenging rate of 61.25 ± 1.90%. Although the SM group showed a higher degree of hydrolysis and produced the highest peptide contents, its highest DPPH scavenging rate was only 41.72 ± 2.03%. This discrepancy indicates that the hydrolysis products differed between the groups in terms of their type or characteristics [28].

### 3.3. Effect of Lipid on Peptide Profile of Different Samples Determined by LC-MS/MS

**Figure 4:**
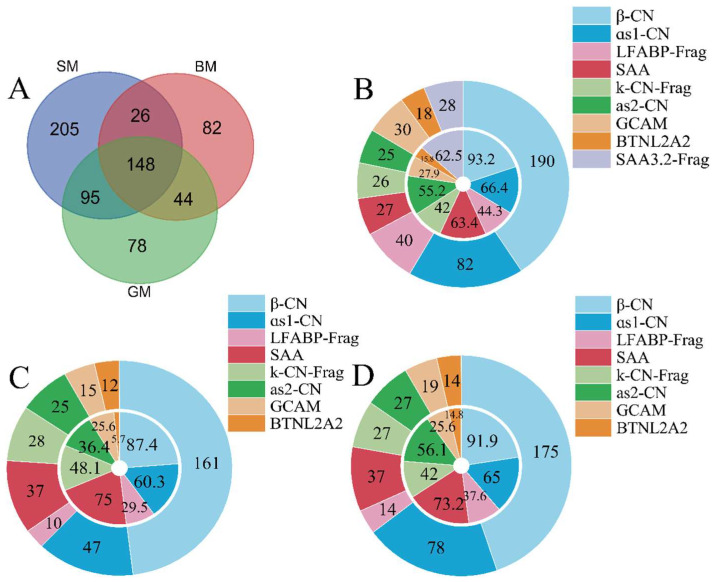
(**A**) Venn diagram of identified peptide numbers from different goat milk emulsions. (**B**–**D**) In vitro digestion peptide coverage of different milk emulsions. The inner ring represents peptide coverage (%), and the outer ring represents peptide number. (**B**) SM group; (**C**) BM group; (**D**) GM group.

The identification of peptides in simulated infant gastric digestion samples was performed using LC-MS/MS. A total of 474, 300, and 365 peptides were identified in the SM, BM, and GM groups, respectively (Figure 4A). Notably, significant differences in peptide types were observed among different groups, with 205, 82, and 78 unique peptides identified in the SM, BM, and GM groups, respectively. This finding suggests that lipids with different properties modulate the enzyme cleavage sites, thereby generating distinct peptide profiles. Upon absorption to the oil–water interface, proteins undergo partial loss of tertiary structure and significant conformational rearrangement. As noted by Zhai et al. [29], the extent of interfacial structure perturbations is governed by the physicochemical properties and attributes of the oil phase. Compared with bovine milk lipid and goat milk lipid, soybean oil, which is rich in long-chain unsaturated fatty acids, shows distinct interfacial and hydrophobic characteristics, leading to different conformational changes of proteins at the oil–water interface. The relatively compact conformation induced by milk lipids reduces protease accessibility, thereby increasing resistance to digestion. In contrast, the interfacial environment formed by soybean oil favors a more extended protein structure, which facilitates protease recognition and hydrolysis, resulting in the generation of more peptides. This matrix-dependent proteolysis is further supported by the work of Maldonado et al. [30], who observed the proteolytic patterns of proteins across different lipid interfaces were substantially altered. Furthermore, protein coverage substantiated that the lipid matrix dictates the degree of proteolysis and protease accessibility. As depicted in Figure 4, peptides derived from β-lactoglobulin and α-lactalbumin remained undetected across all groups, reflecting their inherent digestive resistance [31]. Conversely, for high-abundance proteins, including β-casein (β-CN), αs1-casein (αs1-CN), and κ-casein (κ-CN), the SM group exhibited the broader peptide coverage (*p* < 0.05). This disparity stems from the predominance and flexible, random-coil nature of caseins; unlike globular whey proteins, their structural fluidity facilitates preferential adsorption and extensive unfolding at the oil–water interface. Specifically, the high LCUFA content and unique properties of soybean oil likely exert a pronounced “pulling force” on casein anchors, inducing a conformational transition from a dense, sterically hindered layer into an “accessible-loop” configuration. By lowering the physical barrier for protease engagement and modulating cleavage site exposure, these lipid-specific interfacial environments ultimately reshape the digestible peptide landscape and the potential bioactivity of food proteins.

Among the peptides common to all three groups, 86 bioactive peptides were identified by screening against the BIOPEP database and literature (Table 1). Among these, peptides with dipeptidyl peptidase-IV (DPP-IV) inhibitory activity were the most abundant (45 peptides), consistent with previously reported hypoglycemic effects of goat milk hydrolysates [32]. Additionally, angiotensin-converting enzyme (ACE) inhibitory peptides (23 peptides), antimicrobial peptides (9 peptides), and anxiolytic peptides (2 peptides) were identified. These results suggest that in vitro digests of goat milk may exhibit potential in vitro bioactivity in managing metabolic disorders such as hyperglycemia and hypertension.

Lipid-source-specific peptides also displayed distinct bioactive profiles (Tables S1–S3). The SM group harbored the highest number of unique bioactive peptides (45 peptides), primarily comprising antimicrobial peptides (40 peptides) and immunomodulatory peptides (5 peptides). The BM group contained the fewest (12 peptides), whereas the 31 unique peptides identified in the GM group were exclusively associated with immunomodulatory activity.

Bioactive peptides represent a prominent area of research in functional foods. However, most current studies focus on modulating bioactive peptide generation solely by altering protease systems or protein substrates, often overlooking the role of multi-component interactions within complex food matrices. This study demonstrates that lipid–protein interfacial interactions can significantly modify protease cleavage sites, thereby inducing the generation of distinct bioactive peptide profiles. Furthermore, DPPH radical scavenging assays revealed that even when using the same goat milk protein substrate, different lipid sources resulted in significant variations in the antioxidant activity of the hydrolysates. Collectively, these findings underscore the need for future research to place greater emphasis on food component interactions to enable precise enhancement and targeted modulation of the functional activities of the protein hydrolysates.

### 3.4. Effect of Different Lipid Sources on Peptide Absorption in Caco-2 Cells

**Figure 5:**
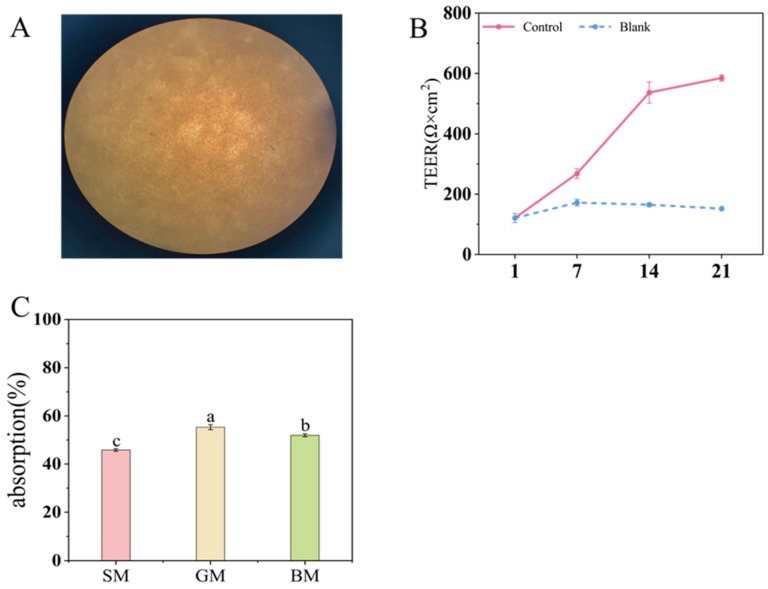
(**A**) Microscopic morphology of Caco-2 cells cultured on transwell plates for 21 days. (**B**) Changes in transepithelial electrical resistance (TEER) of Caco-2 cell monolayers. (**C**) Absorption rate of the polypeptide. (Different letters indicate *p* < 0.05).

Figure 5A demonstrated the microstructure of the monolayer of Caco-2 cells cultured in Transwell plates after 21 days, showing uniform cell distribution with consistent coloration and no significant stratification or aggregation. As shown in 5B, the TEER value reached 590.0 Ω·cm^2^ on day 21, which was significantly higher than that of the blank control (114 Ω·cm^2^, *p* < 0.05), indicating intact cell membrane integrity. Furthermore, the results from the CCK-8 assays demonstrated that the cell viability remained above 80% across all tested concentrations, confirming that CLF and MLF had no significant effect on Caco-2 cell growth [33]. Collectively, these results confirmed the suitability of the model for absorption and functional analyses.

Our in vitro DPPH radical scavenging assay demonstrated that goat milk emulsion samples with different lipid sources exhibited high antioxidant capacity, and peptidomics analysis identified a large number of bioactive peptides. However, for these bioactive substances to exert their potential effects in adults or infants, they must be absorbed into the bloodstream in an active form across the gastrointestinal tract. Intestinal absorption of peptides is a complex process primarily mediated by peptide transporters on the intestinal epithelial cell membrane, the paracellular route, and transcytosis [33,34]. A Caco-2 cell monolayer model cultured for 21 days was employed to investigate peptide transport. As shown in Figure 5C, the GM group exhibited the highest peptide absorption rate (*p* < 0.05), reaching 55.34 ± 1.05%, which was significantly higher than that of the BM (51.22 ± 0.61%) and SM (45.82 ± 0.53%) groups. Molecular weight (MW) characteristics of peptides play an important role in their absorption, with dipeptides and tripeptides demonstrating higher transport efficiency compared to peptides of other lengths. While LC-MS/MS analysis identified fewer peptide species in the GM group than in the SM group, the inherent detection limit of this technique for fragments under three residues suggests a higher prevalence of short-chain peptides in the GM hydrolysates. Conversely, the SM group had a greater number of longer peptides (Figure S1), resulting in higher DH but lower absorption rate. Intestinal absorption is a crucial prerequisite for evaluating bioavailability, and the higher absorption rate of the GM group suggests that it possesses relatively higher bioaccessibility. Previous studies have largely focused on the effects of lipids with different physicochemical properties on protein digestion [19,35]. Although both our research and existing studies indicate that soybean oil promotes the hydrolysis of goat milk proteins [12], we further discovered that different lipid sources influence cleavage sites and lead to divergent bioaccessibility during absorption. Notably, despite soybean oil enhanced the hydrolysis of goat milk proteins, the hydrolyzed group containing animal milk fat exhibited stronger antioxidant activity and a higher absorption rate. Although the specific antioxidant sequences remain to be experimentally validated beyond BIOPEP-based predictions, this study confirmed that lipid–protein interactions fundamentally reshape the peptide landscape. In future studies, the combination of virtual screening and experimental validation to identify key active components would clarify the specific effects of different fats on cleavage sites. A deeper understanding of how interactions between different milk proteins and lipids affect protein and lipid digestion will contribute to the development of higher-quality infant formula.

### 3.5. Effects of Different Hydrolysates on Oxidative Stress of Caco-2 Cells in Intestine

**Figure 6:**
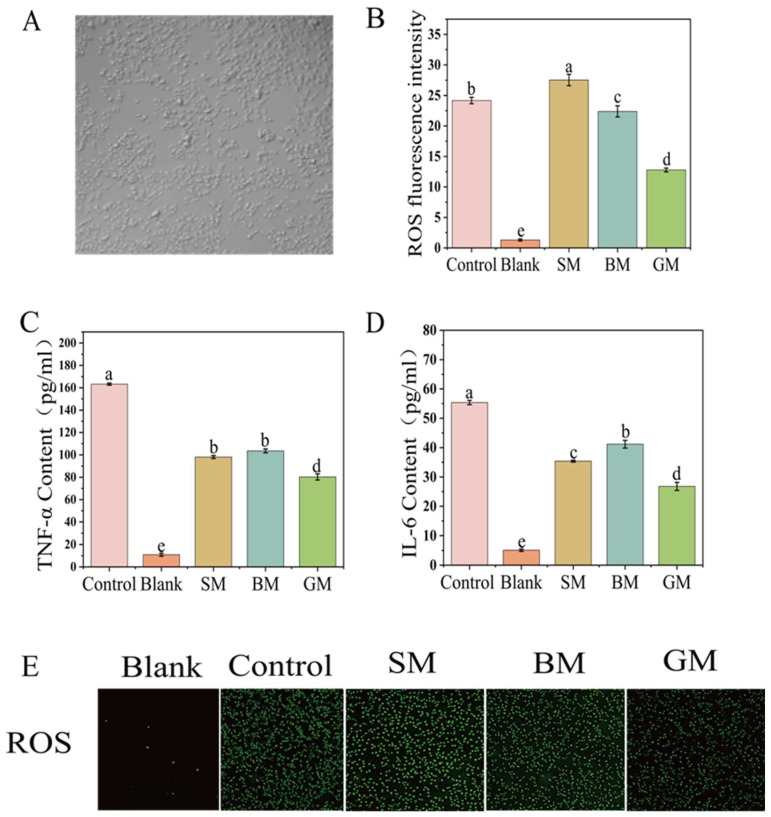
(**A**) Cell growth status induced by 600 μmol/L H_2_O_2_. (**B**) Fluorescence intensity of ROS production in Caco-2 cells. (**C**) Content of TNF-α in Caco-2 cells. (**D**) Content of IL-6 in Caco-2 cells. (**E**) Fluorescence microscopic images of ROS production in cells treated with different goat milk hydrolysates. (Different letters indicate *p* < 0.05).

The intestinal protective efficacy of bioactive peptides is closely related to their structural composition, intestinal absorption efficiency, and synergistic interactions with other nutrients. An H_2_O_2_-induced oxidative stress model of Caco-2 cells was established to evaluate the protective effects of hydrolysates derived from different lipid-containing goat milk emulsions. Figure 6A presents the fluorescence microscopy image showing the impact of different goat milk formulations on the ROS activity in cells. As shown in Figure 6B, the Blank group exhibited relatively low intracellular ROS levels in Caco-2 cells under normal culture conditions. After H_2_O_2_ treatment, the ROS content significantly increased. In the control group, the fluorescence intensity was 24.178. With intervention by goat milk hydrolysate, the ROS levels in the GM group reduced by 50%, which was significantly lower compared with the SM and BM groups (*p* < 0.05), demonstrating the highest resistance to oxidant stress.

Excessive ROS can induce lipid peroxidation, disrupt membrane integrity, and trigger inflammatory signaling cascades. These processes promote the release of inflammatory mediators and chemokines. In addition, inflammatory factors can also promote ROS generation, forming a positive feedback loop to exacerbate cellular damage. IL-6 and TNF-α are 2 typical proinflammatory cytokines in the development of acute and chronic inflammation [36]. The IL-6 and TNF-α concentrations were quantified by Enzyme-linked immunosorbent assays in Caco-2 cell supernatants following H_2_O_2_ exposure and hydrolysate treatment. As shown in Figure 6C,D, the expression of TNF-α and IL-6 significantly decreased in all groups after treatment with different hydrolysis of formula goat milk (*p* < 0.05). Notably, the GM group exhibited significantly lower expression levels of both pro-inflammatory factors compared to the SM and BM groups (*p* < 0.05), indicating that the hydrolysis of GM effectively prevents H_2_O_2_-induced oxidative stress. The hydrolysate from the GM group exhibited higher DPPH radical scavenging activity, peptide absorption rates and a greater abundance of immunomodulatory peptides, which may further suppress inflammatory mediator expression [37]. In addition, bovine and goat milk lipids are rich in branched-chain fatty acids (BCFAs), which may exert anti-inflammatory effects by inhibiting the activation of the NF-κB signaling pathway and the expression of proinflammatory factors [38], thereby further enhancing the protective capacity in intestinal epithelial cells. Although the SM group generated a higher number of antioxidant peptides, its lower peptide absorption rate and the generation of lipid peroxides paradoxically promoted the expression of ROS and the proinflammatory cytokine IL-6. Moreover, high proportions of omega-6 fatty acids in soybean oil have been reported to be associated with proinflammatory responses when consumed excessively [39].

The simulated digestion model and Caco-2 cell system are commonly used in vitro tools for the assessment of intestinal digestion, absorption, antioxidant, and anti-inflammatory activities. However, these models possess notable limitations that warrant critical consideration. The Caco-2 cell line is derived from adult human colorectal adenocarcinoma and only mimics the intestinal barrier of adults, thereby failing to capture the immature structure, permeability, and physiological features of the infant gut. Moreover, the digestion model used in our investigation does not incorporate dynamic gastrointestinal conditions or microbiota interactions. Therefore, further in vivo studies using infant-relevant animal models are required to validate the current findings.

## 4. Conclusions

This study demonstrates that the lipid sources in goat-milk-based formula critically governs protein digestion kinetics, peptide bioaccessibility, and intestinal oxidative stress responses. By integrating in vitro digestion and Caco-2 cell model, we elucidate the divergent impacts of soybean oil (SM), bovine milk fat (BM), and goat milk fat (GM) on the gastrointestinal fate of milk proteins. Our findings reveal a significant decoupling between the degree of proteolysis and subsequent bioaccessibility. While SM group induced the most extensive cleavage sites and highest types of peptide yield, these peptides exhibited limited bioaccessibility with an absorption rate of 45.82 ± 0.53%. In contrast, the homologous GM matrix- achieved a significantly higher absorption rate (55.34 ± 1.05%) than either SM or BM (*p* < 0.05), due to a moderate amount of short-chain fatty acids and unsaturation degree leading to divergent lipid–protein interaction and digestive peptide profiles.

Furthermore, GM-derived digesta conferred pronounced protection against oxidative stress, attenuating reactive oxygen species (ROS) accumulation and downregulating pro-inflammatory mediators TNF-α and IL-6 in H_2_O_2_-induced Caco-2 cells compared to other groups (*p* < 0.05). Collectively, these findings establish that the quality of the peptide profile, governed by lipid-induced interfacial protein conformations, dictates functional outcomes more decisively than the absolute extent of hydrolysis. This work advances our understanding of lipid–protein interplays within milk matrices and advocates for the rational design of lipid matrices to optimize both nutritional delivery and intestinal homeostasis in early life nutrition. Nevertheless, our findings are limited to an in vitro experimental system and may not fully reflect physiological conditions in vivo. Thus, future studies are warranted to verify the actual effects of different lipid sources on infant digestion, absorption, and protective effects in living organisms.

## Figures and Tables

**Table 1:** Common bioactive peptide fragments identified in gastrointestinal digests of various goat milk samples. (Bold text indicates the core active center sequence within the identified peptide fragment).

Common Peptide Fragments	Sequence	Bioactivity	Protein
LYQEP**VLGP**VR	VLGP	DPP-IV Inhibitory	β-CN
**REQEELNV**VGE	REQEELNV	Antimicrobial	β-CN
**LHLPLPL**VQSW	LHLPLPL	ACE-inhibitor	β-CN
**GVPKVKETMVPK**	GVPKVKETMVPK	Antimicrobial	β-CN
V**LPVPQ**KVVPQR	LPVPQ	DPP-IV Inhibitory	β-CN
LLYQEP**VLGP**VR	VLGP	DPP-IV Inhibitory	β-CN
SLSQP**KVLPVPQ**K	KVLPVPQ	ACE inhibitor	β-CN
**QEPVLGPVRGPFP**I	QEPVLGPVRGPFP	ACE inhibitor	β-CN
LSLSQP**KVLPVPQ**K	KVLPVPQ	ACE inhibitor	β-CN
**YQEPVLGPVRGPF**P	YQEPVLGPVRGPF	DPP-IV Inhibitory	β-CN
L**YQEPVLGPVRGPF**	YQEPVLGPVRGPF	DPP-IV Inhibitory	β-CN
TLTDVEK**LHLPLPL**	LHLPLPL	ACE-inhibitor	β-CN
AFLLYQEP**VLGP**VR	VLGP	DPP-IV Inhibitory	β-CN
**YQEPVLGPVRGPF**PI	YQEPVLGPVRGPFPI	Antimicrobial	β-CN
TDVEK**LHLPLPL**VQS	LHLPLPL	ACE-inhibitor	β-CN
**YQEPVLGPVRGPFPI**L	YQEPVLGPVRGPFPI	Antimicrobial	β-CN
L**YQEPVLGPVRGPFPI**	YQEPVLGPVRGPFPI	Antimicrobial	β-CN
LL**YQEPVLGPVRGPF**P	YQEPVLGPVRGPF	Antimicrobial	β-CN
SQPKVLPVPQKVVPQR	KVLPVPQ	ACE inhibitor	β-CN
TDVEK**LHLPLPL**VQSW	LHLPLPL	ACE-inhibitor	β-CN
LTDVEK**LHLPLPL**VQSW	LHLPLPL	ACE-inhibitor	β-CN
SLSQP**KVLPVPQ**KVVPQR	KVLPVPQ	ACE inhibitor	β-CN
LL**YQEPVLGPVRGPFPI**L	YQEPVLGPVRGPFPI	Antimicrobial	β-CN
**PVVVPPFLQPE**IMGVPKVK	PVVVPPFLQPE	Anti-microbial	β-CN
TLTDVEK**LHLPLPL**VQSW	LHLPLPL	ACE-inhibitor	β-CN
LL**YQEPVLGPVRGPFPI**LV	YQEPVLGPVRGPFPI	Antimicrobial	β-CN
LSLSQP**KVLPVPQ**KVVPQR	KVLPVPQ	ACE inhibitor	β-CN
LTDVEK**LHLPLPL**VQSWM	LHLPLPL	ACE-inhibitor	β-CN
**YPVEPF**TESQSLTLTDVEK	YPVEPF	DPP-IV Inhibitory	β-CN
**YPVEPF**TESQSLTLTDVEK	YPVEPF	DPP-IV Inhibitory	β-CN
DMPIQAFLLYQEP**VLGP**VR	VLGP	DPP-IV Inhibitory	β-CN
TLTDVEK**LHLPLPL**VQSWM	LHLPLPL	ACE-inhibitor	β-CN
**YPVEPF**TESQSLTLTDVEKL	YPVEPF	DPP-IV Inhibitory	β-CN
**HKEMPFPKYPVEPFTESQ**SLTL	HKEMPFPKYPVEPFTESQ	DPP-IV Inhibitory	β-CN
P**LTQTPVVVPPF**LQPEIMGVPKVK	LTQTPVVVPPF	ACE inhibitor	β-CN
DMPIQAFLL**YQEPVLGPVRGPFPI**	YQEPVLGPVRGPFPI	Antimicrobial	β-CN
**REQEELNV**VGETVESLSSSEESITH	REQEELNV	Antimicrobial	β-CN
EMPFPK**YPVEPF**TESQ**S**LTLTDVEK	YPVEPF	DPP-IV Inhibitory	β-CN
EMPFPK**YPVEPF**TESQ**S**LTLTDVEKL	YPVEPF	DPP-IV Inhibitory	β-CN
**HKEMPFPKYPVEPFTESQ**SLTLTDVEK	HKEMPFPKYPVEPFTESQ	DPP-IV Inhibitory	β-CN
YPVEPFTESQSLTLTDVEK**LHLPLPL**VQ	LHLPLPL	ACE-inhibitor	β-CN
YPVEPFTESQSLTLTDVEK**LHLPLPL**VQS	LHLPLPL	ACE-inhibitor	β-CN
**HKEMPFPKYPVEPFTESQ**SLTLTDVEKL	HKEMPFPKYPVEPFTESQ	DPP-IV Inhibitory	β-CN
MHQPPQPLSPTVMFPPQSVLSLSQP**KVLPVPQ**K	KVLPVPQ	ACE inhibitor	β-CN
VKETMVPK**HKEMPFPKYPVEPFTESQ**SLTLTDVEK	HKEMPFPKYPVEPFTESQ	DPP-IV Inhibitory	β-CN
MHQPPQPLSPTVMFPPQSVLSLSQP**KVLPVPQ**KVVPQR	KVLPVPQ	ACE inhibitor	β-CN
V**LPVPQ**KAVPQR	LPVPQ	DPP-IV Inhibitory	β-CN
SLSQP**KVLPVPQ**KAVPQR	KVLPVPQ	ACE inhibitor	β-CN
LSLSQP**KVLPVPQ**KAVPQR	KVLPVPQ	ACE inhibitor	β-CN
VLN**ENLLRF**	ENLLRF	ACE inhibitor	α_S1_-CN
**YLGYLEQLLR**	YLGYLEQLLR	Anxiolytic	α_s1_-CN
**YLGYLEQLLR**L	YLGYLEQLLR	Anxiolytic	α_s1_-CN
**SDIPNPIGSE**NSGK	SDIPNPIGSE	Antidiabetic	α_s1_-CN
LSPEVLN**ENLLRF**	ENLLRF	ACE inhibitor	α_S1_-CN
IAVNQELAYF**YP**QLFR	YP	DPP-IV Inhibitory	Lactotransferrin
TDAPSF**SDIPNPIGSE**NSGK	SDIPNPIGSE	Antidiabetic	α_S1_-CN
YTDAPSF**SDIPNPIGSE**NSGK	SDIPNPIGSE	Antidiabetic	α_S1_-CN
QYTDAPSF**SDIPNPIGSE**NSGK	SDIPNPIGSE	Antidiabetic	α_S1_-CN
TQYTDAPSF**SDIPNPIGSE**NSGK	SDIPNPIGSE	Antidiabetic	α_S1_-CN
GTQYTDAPSF**SDIPNPIGSE**NSGK	SDIPNPIGSE	Antidiabetic	α_S1_-CN
YLPLGTQYTDAPSF**SDIPNPIGSE**NSGK	SDIPNPIGSE	Antidiabetic	α_S1_-CN
SAEEQLHSMKEGNPAHQKQPMIAVNQELAYF**YP**QLF	YP	DPP-IV Inhibitory	Lactotransferrin
SAEEQLHSMKEGNPAHQKQPMIAVNQELAYF**YP**QLFR	YP	DPP-IV Inhibitory	Lactotransferrin
DE**VR**KDSKADQFANEWG	VR	DPP-IV Inhibitory	Serum albumin
DQ**VR**EDTKADQF	VR	DPP-IV Inhibitory	Serum albumin
DQ**VR**EDTKADQFANEWG	VR	DPP-IV Inhibitory	Serum albumin
DQ**VR**EDTKADQFANEWGR	VR	DPP-IV Inhibitory	Serum albumin
GMTRDQ**VR**EDTKADQFANEWGR	VR	DPP-IV Inhibitory	Serum albumin
LPYPYYAKPVA**VR**	VR	DPP-IV Inhibitory	Serum albumin
**YP**SYGLNYYQQRPVA	YP	DPP-IV Inhibitory	Lactotransferrin
**INNQFLPYPY**YAKPVAVR	INNQFLPYPY	DPP-IV Inhibitory	κ-CN
DERFFDDKIAKY**IP**IQY	IP	DPP-IV Inhibitory	Serum albumin
VLSR**YP**SYGLNYYQQRPVA	YP	DPP-IV Inhibitory	Lactotransferrin
**INNQFLPYPY**YAKPVAVRSPAQTL	INNQFLPYPY	DPP-IV Inhibitory	κ-CN
**IP**IQYVLSRYPSYGLNYYQQRPVAL	IP	DPP-IV Inhibitory	Serum albumin
QQRPVAL**INNQFLPY**PYYAKPVAVRSPAQTLQ	INNQFLPYPY	DPP-IV Inhibitory	κ-CN
TQPKTNAIPY**VR**	VR	DPP-IV Inhibitory	Serum albumin
AMKPWTQPKTNAIPY**VR**	VR	DPP-IV Inhibitory	Serum albumin
**LP**LSILKEKQLR	LP	DPP-IV Inhibitory	Serum albumin
QNQNPK**LP**LSILK	LP	DPP-IV Inhibitory	Serum albumin
RQPQNQNPK**LP**LSILK	LP	DPP-IV Inhibitory	Serum albumin
SPRQPQNQNPK**LP**LSILKEK	LP	DPP-IV Inhibitory	Serum albumin
VSLVEDHIAEGSVA**VR**	VR	DPP-IV Inhibitory	Serum albumin
GRVSLVEDHIAEGSVA**VR**	VR	DPP-IV Inhibitory	Serum albumin
RIIKDGGIDPL**VR**	VR	DPP-IV Inhibitory	Serum albumin
GLNFDVSLEVSQDPAQASDAHI**YP**VDVGR	YP	DPP-IV Inhibitory	Lactotransferrin
ADDSDPVGGEFLAEGGG**VR**	VR	DPP-IV Inhibitory	Serum albumin
ADDSDPVGGEFLAEGGG**VR**	VR	DPP-IV Inhibitory	Serum albumin
IQR**IP**EVQVYSR	IP	DPP-IV Inhibitory	Serum albumin
I**IP**SAEDPSQDIVER	IP	DPP-IV Inhibitory	Serum albumin
E**IP**LSPMGEDSASGDIETLHS	IP	DPP-IV Inhibitory	Serum albumin

## Data Availability

The original contributions presented in this study are included in the article/Supplementary Materials. Further inquiries can be directed to the corresponding author.

## Supplementary Materials

The following supporting information can be downloaded at: https://www.mdpi.com/article/10.3390/foods15071200/s1, Table S1: Unique bioactive peptide in SM; Table S2: Unique bioactive peptide in BM; Table S3: Unique bioactive peptide in GM. Figure S1: Distribution of peptide lengths in SM, BM and GM groups after in vitro digestion.

## Abbreviations

SM: Skimmed goat milk supplemented with soybean oil; GM: Skimmed goat milk supplemented with goat milk fat; BM: Skimmed goat milk supplemented with bovine milk fat; DPP-IV: Dipeptidyl peptidase-IV; ACE: Angiotensin-converting enzyme; ANOVA: Analysis of variance; CCK-8: Cell counting kit-8; Caco-2: Human colon adenocarcinoma cells; DPPH: 2,2-Diphenyl-1-picrylhydrazyl; ELISA: Enzyme-linked immunosorbent assay; FBS: Fetal bovine serum; IL-6: Interleukin-6; LC-MS/MS: Liquid chromatography-tandem mass spectrometry; PBS: Phosphate-buffered saline; ROS: Reactive oxygen species; SGF: Simulated gastric fluid; SIF: Simulated intestinal fluid; TEER: Transepithelial electrical resistance; TNF-α: Tumor necrosis factor-α; DMEM: Dulbecco’s Modified Eagle Medium; β-CN: β-casein; αs_1_-CN;Alpha-s1-casein; κ-CN: Kappa-casein; MASCOT: Matrix Science Mascot; BIOPEP: BIOPEP-UWM Database of Bioactive Peptides; CLSM: Confocal laser scanning microscope; EDTA: Ethylenediaminetetraacetic acid; He-Ne laser: Helium–neon laser; BCFAs: Branched-chain fatty acids.
