# Supplementary material for: Impact of Lipid Source on Protein Digestion and Absorption in Skimmed Goat Milk and Associated Intestinal Oxidative Stress Responses in a Caco-2 Cell Model
Source: Foods. 2026 Apr 2;15(7):1200. doi: 10.3390/foods15071200 (PMC13072864; doi:10.3390/foods15071200)
Supplement: Supplementary file 1 [file foods-15-01200-s001.zip › foods-4219373-supplementary.pdf]

Table S1 Unique bioactive peptide in SM

Sequence	Bioactivity	Protein	Peptide count
VLVLDTDYKK	Immunomodulatory	Lipocalin/cytosolic fatty-acid binding domain-containing protein	5
SDISLLDAQSAPLR	Antibacteria	Lipocalin/cytosolic fatty-acid binding domain-containing protein	21
ASDISLLDAQSAPLR	Antibacteria	Lipocalin/cytosolic fatty-acid binding domain-containing protein	19

Table S2 Unique bioactive peptide in BM

Sequence	Bioactivity	Protein	Peptide count
EVLNENLLRF	Immunomodulatory	α_{s1}-CN	3
IASAEPTVHSTPTTE	Anti-memory loss	k-CN	1
GPIVLNPWDQVKR	Antioxidant	α_{s2}-CN	3
MKPWTQPKTNAIPYVRYL	Antibacterial	α_{s2}-CN	3
DQHQKAMKPWTQPKTN AIPYVR	Antibacterial	α_{s2}-CN	2

Table S3 Unique bioactive peptide in GM

Sequence	Bioactivity	protein	Peptide count
LYQEPVLGPVRGP	Immunomodulatory	β-CN	31

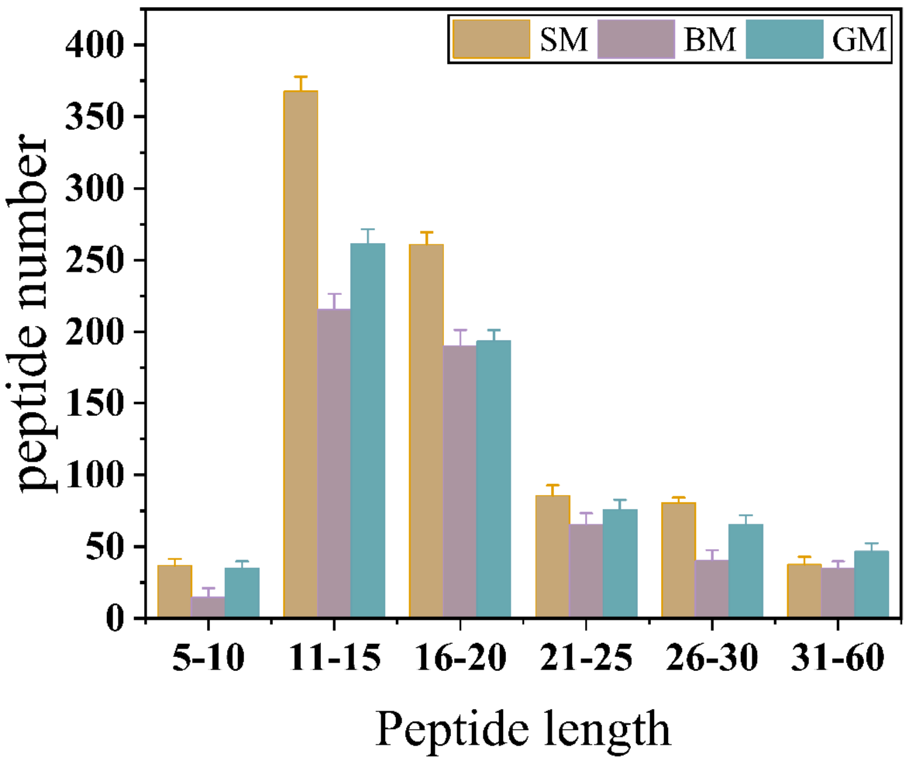

Figure S1. Distribution of peptide lengths in SM, BM and GM groups after in vitro digestion.
